# Metabolic and Genetic Screening of Electromagnetic Hypersensitive Subjects as a Feasible Tool for Diagnostics and Intervention

**DOI:** 10.1155/2014/924184

**Published:** 2014-04-09

**Authors:** Chiara De Luca, Jeffrey Chung Sheun Thai, Desanka Raskovic, Eleonora Cesareo, Daniela Caccamo, Arseny Trukhanov, Liudmila Korkina

**Affiliations:** ^1^Centre of Innovative Biotechnological Investigations (Cibi-Nanolab), Novoslobodskaya Street 36/1, Moscow 127055, Russia; ^2^Active Longevity Clinic “Institut Krasoty na Arbate”, 8 Maly Nikolopeskovsky lane, Moscow 119002, Russia; ^3^Natural Health Farm, 39 Jln Pengacara U1/48, Seksyen U1, Temasya Industrial Park, 40150 Shah Alam, Selangor, Malaysia; ^4^2nd Dermatology Division, Dermatology Institute (IDI IRCCS), Via Monti di Creta 104, 00167 Rome, Italy; ^5^Department of Biomedical Sciences and Morpho-Functional Imaging, Polyclinic University of Messina, 98125 Messina, Italy

## Abstract

Growing numbers of “electromagnetic hypersensitive” (EHS) people worldwide self-report severely disabling, multiorgan, non-specific symptoms when exposed to low-dose electromagnetic radiations, often associated with symptoms of multiple chemical sensitivity (MCS) and/or other environmental “sensitivity-related illnesses” (SRI). This cluster of chronic inflammatory disorders still lacks validated pathogenetic mechanism, diagnostic biomarkers, and management guidelines. We hypothesized that SRI, not being merely psychogenic, may share organic determinants of impaired detoxification of common physic-chemical stressors. Based on our previous MCS studies, we tested a panel of 12 metabolic blood redox-related parameters and of selected drug-metabolizing-enzyme gene polymorphisms, on 153 EHS, 147 MCS, and 132 control Italians, confirming MCS altered (*P* < 0.05–0.0001) glutathione-(GSH), GSH-peroxidase/S-transferase, and catalase erythrocyte activities. We first described comparable—though milder—metabolic pro-oxidant/proinflammatory alterations in EHS with distinctively increased plasma coenzyme-Q_10_ oxidation ratio. Severe depletion of erythrocyte membrane polyunsaturated fatty acids with increased **ω**6/**ω**3 ratio was confirmed in MCS, but not in EHS. We also identified significantly (*P* = 0.003) altered distribution-versus-control of the CYP2C19∗1/∗2 SNP variants in EHS, and a 9.7-fold increased risk (OR: 95% C.I. = 1.3–74.5) of developing EHS for the haplotype (null)GSTT1 + (null)GSTM1 variants. Altogether, results on MCS and EHS strengthen our proposal to adopt this blood metabolic/genetic biomarkers' panel as suitable diagnostic tool for SRI.

## 1. Introduction

The term* electromagnetic hypersensitivity *or* electrosensitivity *(EHS) referred to a clinical condition characterized by a complex array of symptoms typically occurring following exposure to electromagnetic fields (EMFs) even below recommended reference levels and is followed by remission through the complete isolation [1, 2]. The most frequently claimed trigger factors include video display units, radio, televisions, electrical installations, extremely low-frequency ranges of electromagnetic fields or radio-frequencies—including the so-called dirty electricity due to poor isolation of electric wires and telephonic lines, wireless devices, and wi-fi—fluorescent lamps and low-energy lights, appliances with motors, photocopiers, microwave transmitters, and high tension power lines (reviewed in [3, 4]). EHS is characterized by a broad range of nonspecific multiple-organ symptoms implying both acute and chronic inflammatory processes, involving mainly skin and nervous, respiratory, cardiovascular, musculoskeletal, and gastrointestinal systems, in most cases self-reported in absence of organic pathological signs except skin manifestations (reviewed in [2, 5]).

Many efforts have been made to determine if a causal relationship between exposure to EMFs and claimed health symptoms does exist and to identify biologically plausible mechanisms underlying this syndrome (for review, see [2, 6, 7]). Despite the growing wealth of evidences gathered both* in vitro* and* in vivo* on animal models, data from human case-control and double-blind trials attempting to correlate EMFs exposure and claimed symptoms, resulted so far controversial [8–10]. Nowadays, wide gaps still exist in understanding EHS, which most often remains neglected by the medical community or confined within the frame of mere psychogenic etiology [11, 12]. In the persistent lack of a proven pathogenetic mechanism for electromagnetic hypersensitivity and of clinical consensus on the few proposed diagnostic and therapeutic approaches hypothesized, no guideline for safe and efficient validated treatments has been made available until now to the patients worldwide [13, 14].

Nevertheless, the number of subjects self-reporting EHS is progressively increasing, especially in European countries [15–17], with symptoms that are often strongly disabling both professionally and socially, motivating patients to leave home and job to find rescue in “electromagnetic pollution-free” environmental settings. Because of the huge socioeconomic impact anticipated for EHS syndrome worldwide, the World Health Organization has devoted considerable attention to EHS, acknowledging this condition and recommending that people self-reporting sensitivities receive a comprehensive health evaluation [18].

Clinical similarities and frequent comorbidity between EHS and the other medically unexplained multisystem conditions of environmental origin, like* multiple chemical sensitivity *(MCS)*, fibromyalgia *(FM)*, chronic fatigue syndrome *(CFS),* sick building syndrome, Persian Gulf War veteran syndrome*, and* amalgam disease*, to which EHS is often associated [19, 20], have induced many authors to hypothesize that these so-called* idiopathic environmental intolerances *(IEI), more extensively also defined as* sensitivity-related illnesses *(SRI) [21], may share common genetic and/or metabolic molecular determinants connected with an impaired capability to detoxify xenobiotics (for review, see [19, 22]). Our group has evidenced for the first time a set of altered metabolic blood parameters—comprising selected redox-active and detoxifying enzymes, low-molecular weight antioxidants and oxidation markers, membrane polyunsaturated fatty acid, and proinflammatory cytokine patterns—specifically and selectively compatible with the MCS condition [23]. Recently, we contributed to the still open issue of possible genetic polymorphic patterns associated with MCS proneness, proposing a pattern of genotypic alterations of the cytochrome P450 isoenzymes CYP2C9, CYP2C19, and CYP2D6, as candidate risk factors for this specific condition, also being potentially able to discriminate different environmental-borne hypersensitivities (MCS, FM, and CFS), depending on specific combinations of their mutated alleles [24].

In this study, the working hypothesis was that EHS, as previously proposed for MCS and other environmental SRI [19, 22], may as well be based on aberrant responses to physic or chemical xenobiotic stressors through airborne or other routes of exposure, due to inherited or/and acquired dysfunction of the chemical defensive system, that is the interrelated network of phase I and II xenobiotic-metabolizing and antioxidant enzymes [19]. Based on the results of our past clinical studies on MCS, FM, and CFS, we sought to assess if similar profiles of metabolic or genetic dysfunctions could be found in those subjects self-reporting EHS phenotype. To this purpose, we measured possible alterations of a previously identified panel of twelve blood redox and lipid parameters and frequencies of selected genetic mutated variants of a set of drug-metabolizing enzymes and transcription factors with first-line roles in the detoxification of physical and chemical xenobiotics, in a group of 153 patients self-reporting EHS symptoms, co-morbid in most cases with different degrees of MCS symptoms. Results were compared to those obtained on 147 MCS patients without EHS symptoms and on a healthy control group of 132 age- and sex-matched subjects, all groups enrolled within the Italian population.

## 2. Materials and Methods

### 2.1. Patients

**Figure 1:**
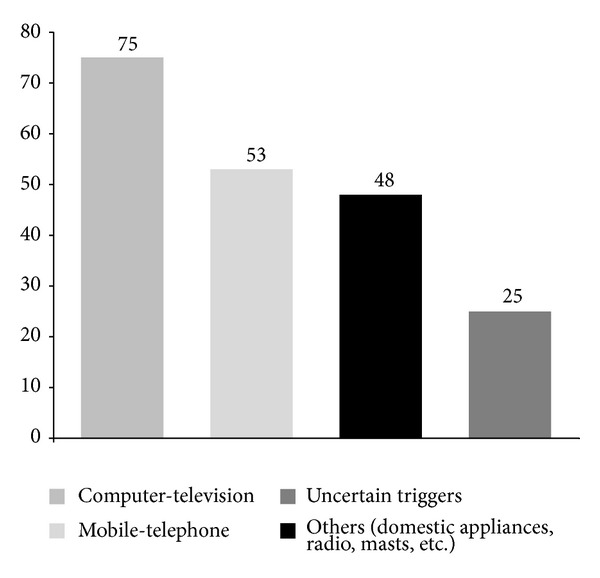
Electromagnetic field sources reported as symptom triggers in the group of patients self-reporting electromagnetic hypersensitivity (EHS, *n* = 153). Data are expressed as percent of patients affected on the total number of patients.

A group of 153 Italian Caucasian consecutive subjects self-reporting hypersensitivity to electro-magnetic fields (EHS group) as described in Figure 1 were enrolled in the study at a specialized Diagnostic Unit for Redox Balance of Istituto Dermopatico dell'Immacolata, IDI IRCCS, Rome, Italy. Age ranged from 16 to 75 years of age (mean ± SD: 46.8 ± 11.7) and female sex represented 85.6% (131 subjects). This group was compared with a size-matched group of 147 patients (age range 19–72 y, mean ± SD: 49.6 ± 12.8, 129F (87.8%)/18M), diagnosed with MCS, but not reporting any symptom of EHS (MCS group). MCS diagnosis was set in both groups according to Cullen's criteria [25] and modified Quick environmental Exposure and Sensitivity Inventory (QEESI) questionnaire scoring [26, 27]. Cullen's criteria refer to a disorder characterized by symptoms that involve more than one organ system and are regularly elicited by chemically unrelated compounds at doses far below those known to cause adverse effects in the general population. Symptoms typically improve considerable or heal completely after trigger withdrawal [25]. QEESI is a validated self-administered questionnaire developed as a screening tool for patients with multiple chemical sensitivity. It is based on five different scales of assessment: symptoms severity, chemical triggers, other triggers, life impact, and finally a masking index to ongoing exposures [26, 27]. A modified QEESI score of 10 common environmental exposures and 10 major symptoms enabled the diagnosis of MCS: full diagnosis (20 ≤ Score ≤ 30) or strongly suspected diagnosis (sMCS, suspected MCS), that is subjects fulfilling diagnostic criteria only partially (10 ≤ Score ≤ 20), or subjects excluded from enrollment (0 ≤ Score ≤ 10) [23]. As commonly seen by our group occurring in the Italian patient population, the large majority (94.7%) of the EHS group was also affected with multiple chemical sensitivity (fully diagnosed or suspected MCS).

A cohort of 132 healthy age- and sex-matched subjects was enrolled as the control group (CTR group), (age range 18–74 y, mean ± SD: 45.3 ± 12.4, 109F (82.6%)/23M), according to the established criteria of (i) absence of any clinically diagnosed disease, in particular allergic or immunologic disturbances, (ii) no drug or nutraceutical supplement since at least six weeks, at the time of blood sampling, and (iii) whole blood total production of reactive oxygen and nitrogen species (ROS/RNS) below 650 cps/*μ*L, as determined by luminol-dependent chemiluminescent response to phorbol 12-myristate 13-acetate (PMA) [28] (Study protocol approval by Istituto Dermopatico dell'Immacolata—IDI IRCCS, Rome, Italy—Ethical Committee, n.52/CE/2010).

All patients and controls entering the study had taken no drugs or nutraceutical supplements known to interfere with metabolizing/antioxidant enzymes activity since at least six weeks, at the time of blood sampling. Nonsmokers in the patient groups were, respectively, 89.3% in EHS and 81.8% in MCS, and 85.2% in the CTR group; undetermined smoking habits were registered in 2% of EHS and 7% of MCS patients, and in 5% of controls. Patients and controls were selected from different Italian regions in the attempt to minimize the historical genetic variability in this country [29]. Demographic information (age, race, weight, and height) and a detailed medical history were recorded in a standardized questionnaire-assisted interview, by trained medical personnel. In particular, subjects were asked to report age at onset of symptoms, agents or events likely to initiate EHS and MCS condition, if recognized, and those capable of triggering symptoms once the condition was established. No alcohol or drug abusers were present in any of the three cohorts studied.

The study protocol was reviewed and approved by the Hospital Ethical Committee Board (IDI IRCCS n.121/CE/2008). All subjects gave informed consent to personal and anamnestic data collection, blood sampling for the specific sets of analyses, and blood fraction's banking.

### 2.2. Reagents and Assay Kits

Majority of chemical reagents, HPLC standards, mediums, fluorogenic probes, and reverse transcription polymerase chain reaction (RT PCR) primers for gene polymorphism analyses were from Sigma Chemical Co. (St. Louis, MO, USA); kits were from Cayman Chem. Co. (Ann Arbor, MI, USA)—enzyme activities are from Qiagen (Hilden, Germany)—DNA extraction is from Applied Biosystems Inc. (Foster City, CA, USA)—polymerase chain reaction is from PCR Kit for CYPs.

### 2.3. Redox Studies

Complete differential blood cell counts and metabolic/genetic analyses were performed on fresh EDTA-anticoagulated venous blood of 12-hour fasting subjects. Biochemical assays were performed on plasma or erythrocytes (RBC) either immediately (coenzyme Q_10_—CoQ_10_) or within 72 hr. on sample aliquots stored at −80°C under argon. Whole blood luminol-dependent chemiluminescence (CL) response to phorbol 12-myristate 13-acetate (PMA) was quantified by chemiluminescence according to [28], levels of (nitrites/nitrates) by Griess reagent [30]. Plasmatic total antioxidant capacity (TAC) was determined as described previously [31]. Reduced and oxidised glutathione (GSH and GSSG) levels in erythrocytes [32], reduced and oxidized CoQ_10_, and alpha-tocopherol levels in plasma [33] were quantified by HPLC equipped with array photodiode and electrochemical detection. Activities of CuZn superoxide dismutase (CuZn-SOD) [34], catalase [35], glutathione S-transferase (GST) [36], and glutathione peroxidase (GPX) [37] in erythrocytes were measured spectrophotometrically.

### 2.4. Erythrocyte Membrane Fatty Acid Profiling

The fatty acid (FA) pattern of erythrocyte membrane phospholipids was analyzed by gas-chromatography coupled with mass spectrometry with the selected ion monitoring technique, set to identify C16:0, C16:1, C18:0, C18:1*cis*, C18:1*trans*, C18:2*ω*6, C18:3*ω*6, C20:4*ω*6, C20:5*ω*3, C22:4*ω*3, C22:5*ω*3, and C22:6*ω*3 peaks [38]. Results were expressed as percent of the total fatty acid content of membrane phospholipids for saturated + monounsaturated FA (SFA), polyunsaturated FA (PUFA), and single representative FA of the *ω*3 and *ω*6 series.

### 2.5. Genotyping of Drug Metabolism-Related Enzymes

Targeted genotype analysis was performed on subgroups of EHS (*n* = 127) and MCS patients (*n* = 85) and of controls (*n* = 68), with reduced due to financial limitations—but yet representative—group sizes for single genotype. Genomic DNA was purified from 400 *μ*L of human whole blood using the QIAamp DNA Blood Mini Kit (Qiagen, Hilden, Germany) according to the manufacturer's instructions. DNA was quantified spectrophotometrically at 260 nm, aliquoted, and stored at −20°C until being assayed. Genotyping and controls for eight single nucleotide polymorphisms in drug metabolism- and inflammation-related genes were carried out by real-time PCR allelic discrimination using predesigned TaqMan single nucleotide polymorphism (SNP) genotyping assays available from Applied Biosystems (Applera Italia, Monza, Italy). The polymorphisms analyzed were those of genes coding for the following: cytochrome P450 (CYP), family 2, subfamily C, polypeptides 9 and 19, namely, CYP2C9*2 (C>T, rs1799853; assay ID: C_25625805_10), CYP2C9*3 (A>C, rs1057910; assay ID: C_27104892_10), and CYP2C19*2 (G>A, rs4244285; assay ID: C_25986767_70); CYP2 subfamily D, polypeptide 6, namely, CYP2D6*4 (1846G>A, rs3892097; assay ID: C_27102431_D0) and CYP2D6*41 (C>T, rs28371725; assay ID: C_34816116_20); aryl hydrocarbon receptor (AHR) Arg554Lys variant (G>A, rs2066853; assay ID: C_11170747_20). Genotyping reactions were set up in a 96-well plate on a 7900HT fast real-time PCR System (Applied Biosystems, Foster City, CA) and were carried out in a final volume of 20 *μ*L containing 1× TaqMan Genotyping Master Mix, 1× TaqMan-specific assay, and 10 ng genomic DNA, using thermal cycling conditions suggested by manufacturer's protocols.

The GSTP1 polymorphisms resulting in an Ile (wild type) to Val (mutant) substitution at residue 104 in exon 5 and Ala (Wild Type) to Val (mutant) substitution at residue 113 in exon 6 were determined by real time PCR using two different fluorogenic probes for the wild type and the mutant. By combining the results of the analysis of exon 5 and exon 6, the allelic setup was determined (GSTP1*A = Ile104/Ala113; GSTP1*B = Val104/Ala113; GSTP1*C = Val 104/Val113). The deletion polymorphisms for the GSTM1 and the GSTT1 genes were determined simultaneously in a single assay using a multiplex PCR approach with the amplification of the GSTM1 and the GSTT1 genes from genomic DNA and using *β*-globin as internal control [39].

### 2.6. Statistical Analysis

Statistic significance of redox and fatty acid parameters was evaluated using STATISTICA 6.0 program (StatSoft Inc., Tulsa, OK, USA). Normality of data was checked using the Shapiro-Wilk test. Since the distribution of the data in the three groups was significantly different from normal, nonparametric statistics was used. Values were presented as mean, standard error of the mean, and 1.96× standard error. Mann-Whitney* U*-test for independent samples was employed for comparison between case groups and controls. All reported* P* values are from two-tailed tests, and* P* values of less than 0.05 were considered to indicate statistical significance. If necessary,* P* values were adjusted for multiple comparisons using the Bonferroni adjustment.

The comparison of allele and genotype frequencies between patients and controls, or in-between patient cohorts, was performed using the GraphPad Prism 4 software (San Diego, CA, USA). Genotypes frequencies of patients' and control groups were compared with Fisher's exact test. A* P* value ≤0.05 or lower was regarded as statistically significant. Odds ratio (OR) and 95% confidence interval (CI) were used to analyze the frequency of genotypes since they provide a measure of the strength of association, compared to the control population.

## 3. Results

### 3.1. Anamnestic and Lifestyle Data

**Figure 2:**
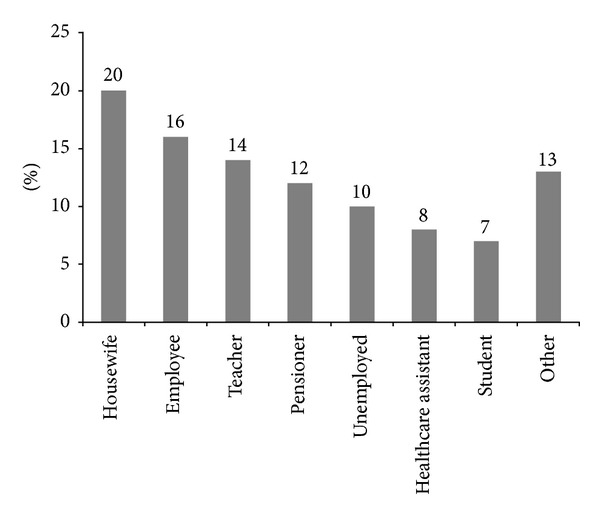
Occupational features in the group of patients self-reporting electromagnetic hypersensitivity (EHS, *n* = 153). Data are expressed as percentage of the total number of patients.

Among EMFs emissions recognized as trigger factors in the group of 153 patients self-reporting electromagnetic hypersensitivity-EHS, video display units and television were the most frequently reported sources (75% of patients), followed by mobile and landline phones (53%) and by domestic appliances (48%), while 25% of the electrosensitive population studied could not indicate a specific triggering factor (Figure 1). Potential exposure patterns to indoor EMFs can be inferred from the analysis of the percent distribution of occupational features in the EHS group, described in Figure 2.

**Figure 3:**
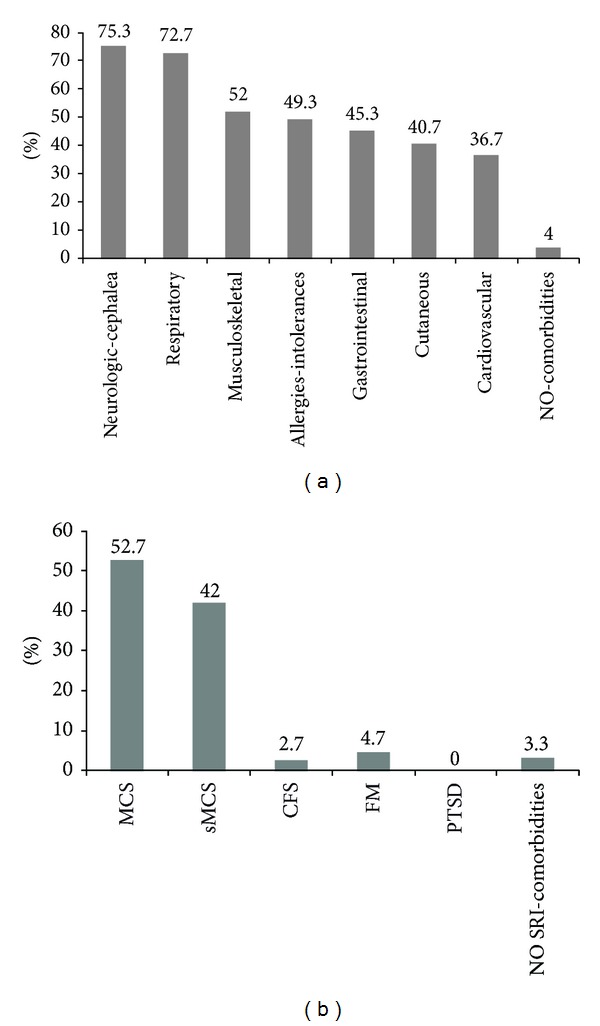
Distribution of specific organ comorbidities (a) and sensitivity-related illness-SRI comorbidities (b) registered in the case history of the group of patients self-reporting electromagnetic hypersensitivity (EHS, *n* = 153). Data are expressed as percentage of the total patient group, for patients affected by each single category of organ pathologies (a), and by each SRI (b), specifically multiple chemical sensitivity (MCS) or suspected MCS (sMCS), chronic fatigue syndrome (CFS), fibromyalgia (FM), and posttraumatic stress disorders (PTSD).

The percent distribution of concomitant organ diseases (comorbidities) in the EHS patient cohort, as obtained by clinical anamnestic evaluation, is presented in Figure 3(a). Body mass index (BMI) in the EHS subjects ranged between 15 and 37 (mean ± SD: 23.3 ± 5.06), while in the group of MCS without electro-hypersensitivity there were 20% overweight patients (BMI: 25.00–29.99), 11% obese (BMI: 30.00–34.99), 2% severely obese (BMI: 35.00–39.99), 11% underweight (BMI: 18.49–16.00), and only 56% normal-weight patients (BMI: 18.50–24.99).  Figure 3(b) shows the percent distribution of the other sensitivity-related illness-SRI coexisting with electromagnetic hypersensitivity in the EHS study cohort, where the 52.7% of MCS cases and the 42% of suspected MCS cases sum up clearly predominant 94.7% of multiple chemical sensitivity symptomatic subjects, within the patients self-reporting EHS symptoms.

**Figure 4:**
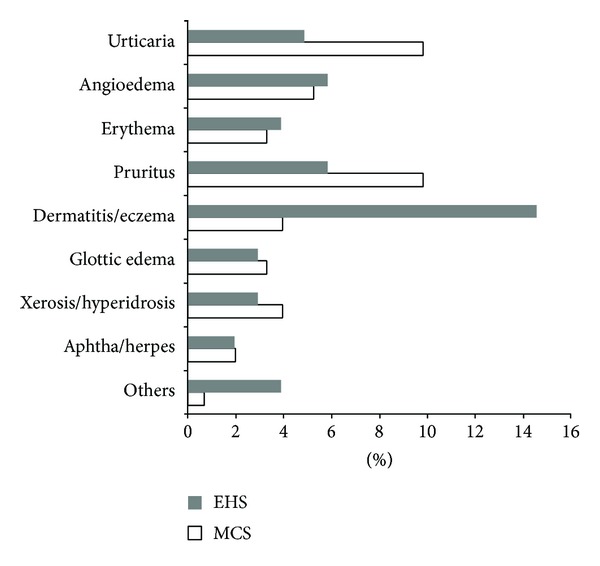
Skin manifestations (common symptoms and specific diseases) registered in the case histories of the groups of patients self-reporting electromagnetic hypersensitivity (EHS, *n* = 153) and of patients affected by multiple chemical sensitivity without EHS symptoms (MCS, *n* = 147). Data are expressed as percentage of patients affected by each specific class of cutaneous manifestations.

In Figure 4, the main classes of cutaneous symptoms or specific diseases recorded by the clinical operators through questionnaire-assisted anamnestic interview are represented, evidencing remarkable prevalence of acute dermatitis or chronic eczema conditions (both symptoms referable to different etiologies) among EHS subjects, whilst in the MCS group without electro-hypersensitivity urticaria and itching referable to (different etiologies) represented the most common findings.

### 3.2. Blood Metabolic Parameters

**Figure 5:**
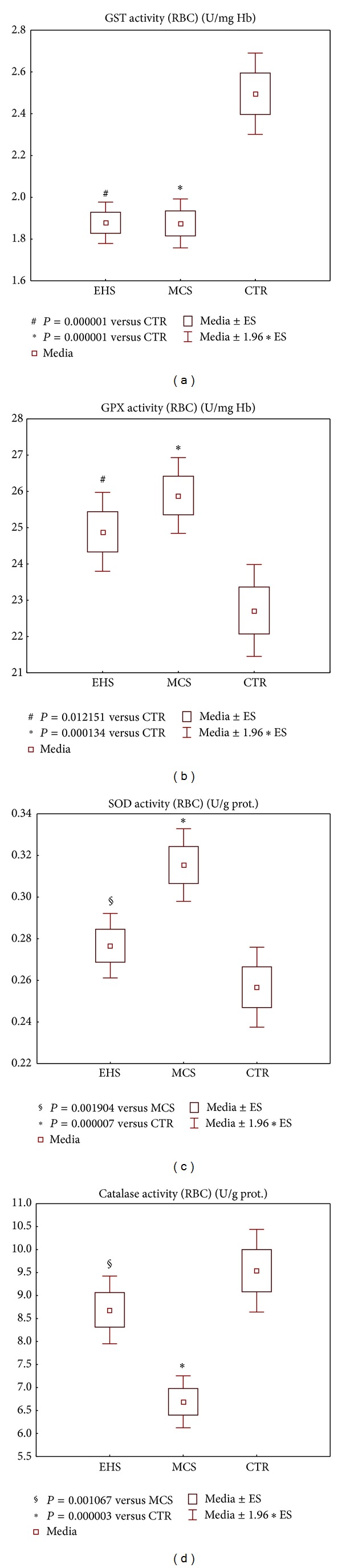
Metabolic redox parameters: the antioxidant/detoxification enzymatic activities of erythrocyte GST (a), GPX (b), CuZnSOD, (c) and catalase (d), in the groups of patients self-reporting electromagnetic hypersensitivity (EHS, *n* = 153), of patients affected by multiple chemical sensitivity without EHS symptoms (MCS, *n* = 147), and of control healthy subjects (CTR, *n* = 132). Values are represented as mean (□), standard error of the mean (upper and lower limits of the box), 1.96× standard error (upper and lower whiskers). Intergroup significant differences (*P*) are reported under each panel. RBC: red blood cells; SOD (CuZn superoxide dismutase); GST: glutathione S-transferase; GPX: glutathione peroxidase; prot.: proteins; Hb: haemoglobin.

**Figure 6:**
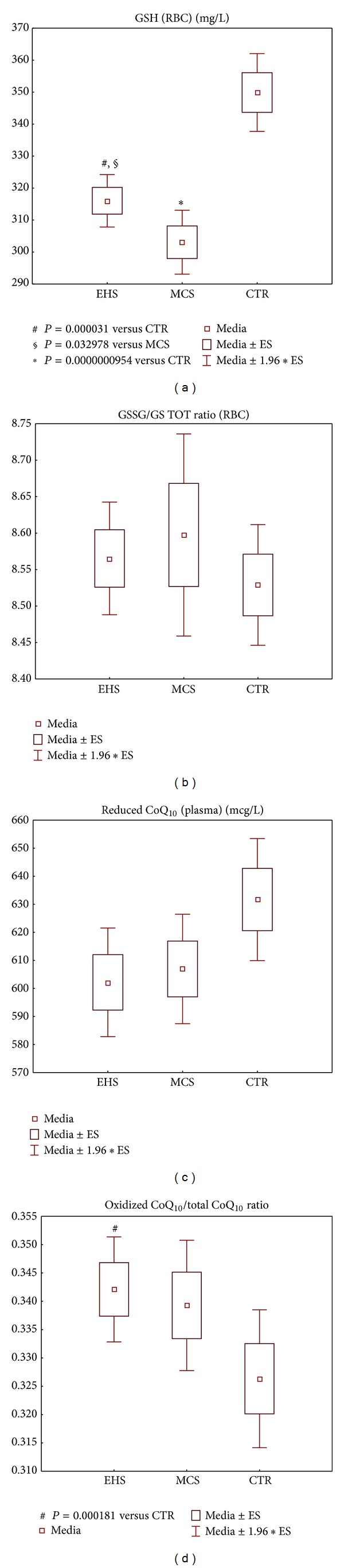
Metabolic redox parameters: levels of the low-molecular weight antioxidants/cofactors, erythrocyte glutathione ((a) and (b)), and plasma coenzyme Q_10_ ((c) and (d)), in the groups of patients self-reporting electromagnetic hypersensitivity (EHS, *n* = 153), of patients affected by multiple chemical sensitivity without EHS symptoms (MCS, *n* = 147), and of control healthy subjects (CTR, *n* = 132). Values are represented as mean (□), standard error of the mean (upper and lower limits of the box), and 1.96× standard error (upper and lower whiskers). Intergroup significant differences (*P*) are reported under each panel. RBC: red blood cells: GSH: glutathione reduced form; GSSG: glutathione oxidized form; GS TOT: total glutathione; CoQ_10_: coenzyme Q_10_.

**Figure 7:**
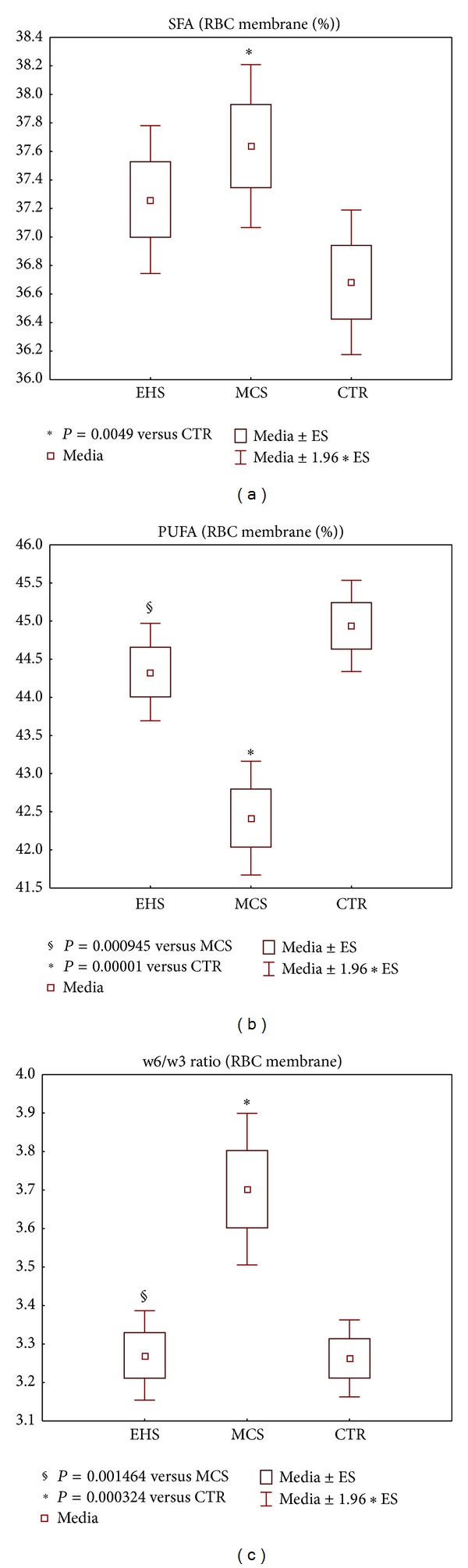
Selected representative parameters describing fatty acid (FA) patterns of erythrocyte membrane phospholipids, in the groups of patients self-reporting electromagnetic hypersensitivity (EHS, *n* = 58), of patients affected by multiple chemical sensitivity without EHS symptoms (MCS, *n* = 54), and of control healthy subjects (CTR, *n* = 70). (a) % saturated and monounsaturated acid (SFA) on total FA content of phospholipids, (b) % polyunsaturated fatty acids (PUFA) on total FA content of phospholipids, and (c) ratio omega-6/omega3 PUFA. Values are represented as mean (□), standard error of the mean (upper and lower limits of the box), and 1.96× standard error (upper and lower whiskers). Intergroup significant differences (*P*) are reported under each panel. RBC: red blood cells.

**Figure 8:**
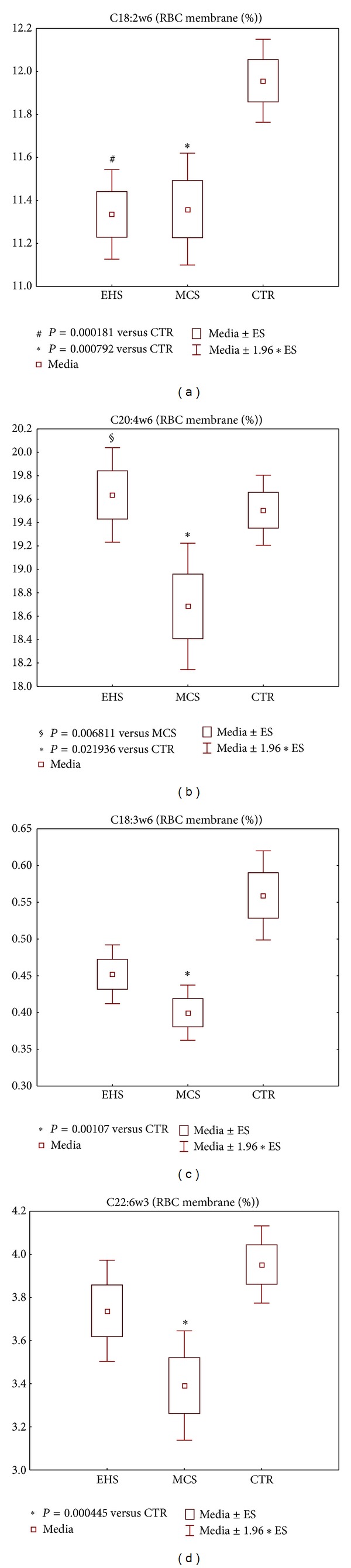
Selected representative omega-6 and omega-3 polyunsaturated fatty acids (PUFA) of erythrocytes membrane phospholipid fatty acids (FA), in the groups of patients self-reporting electromagnetic hypersensitivity (EHS, *n* = 58), of patients affected by multiple chemical sensitivity without EHS symptoms (MCS, *n* = 54), and of control healthy subjects (CTR, *n* = 70). (a) % C18:2*ω*6; (b) C20:4*ω*6; (c) C18:3*ω*6; (d) C22:6*ω*3 FAs, on total FA content of phospholipids. Values are represented as mean (□), standard error of the mean (upper and lower limits of the box), and 1.96× standard error (upper and lower whiskers). Intergroup significant differences (*P*) are reported under each panel. RBC: red blood cells. 18:2*ω*6 (linoleic acid), 18:3*ω*6 (alpha linolenic acid), 20:4*ω*6 (arachidonic acid), and 22:6*ω*3 (docosahexaenoic acid).

Candidate metabolic biomarkers of electrhypersensitivity, as compared to multiple chemical sensitivity without EHS manifestations and to the corresponding values of the same blood parameters in the group of healthy controls, are shown in Figures 5–8.

A set of 12 metabolic enzymatic and nonenzymatic redox parameters were measured in the blood of the 153 EHS patients, 147 patients with MCS reporting no EHS, and in the 132 healthy age- and sex-matched CTR subjects.  Figure 5 shows the respective alterations of all four enzymatic activities studied in the EHS group, compared to MCS and to control values. More specifically, GST activity in erythrocytes was severely decreased in both EHS and MCS groups, compared to the CTR group (*P* < 0.0001), with no significant difference between the patients' subgroups (Figure 5(a)). A clearly uprisen erythrocyte GPX activity was registered in the EHS and more markedly in the MCS groups versus controls (*P* < 0.05 and *P* < 0.001 resp.) (Figure 5(b)), and the same was true for RBC CuZnSOD activity of MCS group versus CTR (*P* < 0.0001), while EHS patients showed only a trend towards increased activity (*P* < 0.05 versus MCS) (Figure 5(c)). Finally, Figure 5(d) shows how catalase activity rate in RBC was found decreased in both EHS and MCS patients as compared to healthy CTR, though reaching a clear-cut and elevated statistical significance only in the MCS group (*P* < 0.0001), as previously already reported [23].

Figure 6 describes the alteration of the blood levels of four redox-active low-molecular weight parameters investigated as suitable biomarkers of EHS condition, in comparison to the uncomplicated MCS and the healthy control study cohorts. The levels of both reduced (GSH) and oxidized (GSSG) glutathione forms (data shown in the figure only for GSH (Figure 6(a))) were strongly decreased in the RBC of EHS and MCS environmentally sensitive groups as compared to CTR subjects (GSH: *P* < 0.0001 for both groups; GSSG: *P* < 0.001 and *P* < 0.0001, resp., for EHS and MCS), although decrease scores for both glutathione forms were inferior in the EHS than in the MCS subgroup (GSH: *P* < 0.05; GSSG: *P* < 0.001 in EHS versus MCS). Also the ratio of GSSG/GSH (Figure 6(b)), indicating the relative oxidation grade of the erythrocyte glutathione marker, displayed a trend to elevation in the two patient subgroups versus control, although data were too scattered to reach any statistical value.

The plasmatic levels of coenzyme Q_10_ and alpha-tocopherol displayed a similar trend-to-depletion in both patient subgroups versus controls.  Figure 6(c) reports results of ubiquinol (CoQ_10_H_2_, the reduced form of coenzyme Q_10_) analysis which, together with levels of total CoQ_10_ (reduced + oxidized forms) and of alpha-tocopherol (both groups of data not shown)—showed similar trend of reduction for EHS as well as MCS subgroups, as compared to CTR group, though lacking statistical significance. Indeed, we found a higher percent coenzyme Q_10_ oxidation (ratio oxidized-CoQ_10_/total-CoQ_10_), significant versus CTR at *P* < 0.001 in EHS patients, not confirmed for MCS patients, as reported in Figure 6(d).

Although a trend-to-increase in the values of whole blood chemiluminescence (CL) and to decreased levels of plasmatic total antioxidant capacity (TAC) were recorded for both patient subgroups compared to controls, differences were unable to reach any statistical significance (data not shown). The increase of  NO_2_^−^/NO_3_^−^  plasma levels of MCS patients obtained in our previous study [23] was not confirmed in this new MCS subgroup, as well as in the EHS group of the present study, respectively, averaging or being inferior to control values (data not shown).

Since the majority of the above metabolic data were similar for EHS and MCS subgroups, the costly and time-consuming analyses of fatty acid profiles were carried out on a more limited subgroup of patients who fully corresponded to all diagnostic criteria. Representative fatty acid profiles in the phosholipid fraction of the erythrocyte membranes of EHS (*n* = 58), MCS (*n* = 54) and CTR (*n* = 70) patients are shown in Figures 7 and 8. The comparative analysis of the fatty acid (FA) profiles in the erythrocyte membranes of the 3 studied groups showed elevated levels of the saturated and monounsaturated fatty acid fraction (SFA) for both environmental-sensitive patients (Figure 7(a)) and correspondingly depleted levels of the polyunsaturated fatty acid fraction (PUFA) (Figure 7(b)), with both parameters statistically significant at *P* < 0.05 for MCS patients versus controls, whilst the EHS group differed sensibly from MCS in displaying only a mild trend-to-alteration of fatty acid patterns versus control group. In detail, the percent levels of the omega-6 FA linoleic (18:2*ω*6), alpha linolenic (18:3*ω*6), arachidonic (C20:4*ω*6), and the omega-3 FA docosahexaenoic (C22:6*ω*3) (Figures 8(a)–8(d)) were lower than control values in both EHS and MCS cohorts, although the clear-cut statistical significance registered for the MCS group (*P* < 0.05–0.001 for all 4 parameters) was confirmed in EHS patients only for linoleic acid fraction (*P* < 0.001) (Figure 8(a)). Finally, the range of the *ω*6/*ω*3 PUFA ratio in electrosensitive subjects practically equalled that of controls, whilst MCS patients showed significantly increased values versus both CTR (*P* < 0.001) and EHS group (*P* < 0.05), as reported in Figure 7(c).

### 3.3. Genetic Parameters

The main results of genotype analysis for a selected panel of detoxifying enzymes, obtained on limited subgroups of EHS, MCS, and controls, are illustrated in Table 1. Having previously demonstrated in the MCS population a significantly higher-versus-CTR frequency of the homozygous mutated *1 allele and a CYP2C19*2 heterozygous genotype *1/*2, with a lower frequency of the *2 allele in the homozygous and heterozygous forms [24], we here confronted the panel of previously investigated CYP isozymes in the EHS versus the already studied MCS cohort previously studied. Genotype frequencies for cytochrome P450 CYP2C19 SNP variants in EHS and MCS patients' groups showed that the CYP2C19*1/*1 and the CYP2C19*1/*2, *2/*2 genotypes differed with statistical significance at *P* = 0.003 between EHS (*n* = 29) and MCS (*n* = 85) groups. The other gene polymorphisms of CYPs studied (CYP2C9 and CYP2D6), as well as the aryl hydrocarbon receptor (AHR) variant Arg554Lys, displayed similar frequency distributions for EHS and MCS patients (data not shown).

Genotype frequencies of the glutathione S-transferase (GST) isoenzymes GSTP1, GSTM1, and GSTT1, previously found not significantly differing in MCS versus healthy control populations [23], were compared in 127 EHS patients versus 68 CTR subjects. No statistically significant differences were observed for GSTP1 in the frequency of the GSTP1*A, GSTP1*B, or GSTP1*C homozygous and heterozygous variants between the EHS patient and control groups (Table 1).

The statistical analysis of the distribution of GSTM1 and GSTT1 isoenzymes showed no statistical difference in homozygous + heterozygous and null genotype variants neither in GSTM1 nor in GSTT1, when analyzed independently. Conversely, the combined GSTM1 (*0/*0) + GSTT1 (*0/*0) null genotypes differed significantly (13% versus 1.5%, resp.), with *P* = 0.007, in EHS patients versus CTR subjects, conferring to this association of gene variants 9.7 times higher risk (OR: 95% C.I. = 1.3–74.5) of developing EHS compared to other GSTM1 and GSTT1 combinations of genotypes examined (Table 1).

## 4. Discussion

Till now, no causal relationship between electromagnetic fields exposure and onset of clinical symptoms has been clearly proven. Nevertheless, the term electric hypersensitivity is currently used both by patients who claim health effects of environmental electromagnetic pollution and doctors to define patient clusters of symptoms [40]. Most of the evidences about altered organic parameters due to EMF exposure have been so far obtained on cell or animal models. Very few human studies investigated possible organic parameters distinctive of the hypersensitivity to electromagnetic stressors ([41, 42]; for review, see [2]).

Main difficulties for clinical studies' implementation arise from the necessity to deal with patients in a protected environment, sheltered from EMF sources and also free of chemical barriers, since the majority of electrosensitive patients are also intolerant to a multiple array of chemical triggers [43]. Indeed, in the group of 153 EHS subjects enrolled for this study, 145 were also affected at different degrees by MCS symptoms (Figure 3(b)). The experimental group of EHS patients was exposed by lifestyle to the most common electromagnetic sources deriving mainly from indoor or outdoor urban electromagnetic pollution and no heavy professional exposure in industrial settings was recorded in the group (Figure 2). In addition, EHS patients shared with MCS patients the sensitivity to the most frequent organic chemical triggers initiating and sustaining MCS.

Another relevant issue complicating human studies is connected with the difficulties encountered in provocation studies, aimed at connecting the electromagnetic trigger with electrohypersensitivity symptoms' onset. These difficulties arise generally from the necessity to standardize types and dosages of EMF sources, from the broad qualitative and quantitative range of individual multiorgan responses to trigger, difficult to measure objectively, and also from heavy psychoemotional bias factors affecting experimental protocols and their repeatability [44, 45]. Notably, provocation studies commonly proposed as the main milestone for EHS assessment and validation are based on the questionable assumption that the individual capability to directly perceive EMFs at low or very low intensities below established toxicological thresholds, claimed by EHS subjects in analogy with MCS odor perception, may be* conditio sine qua non* for EHS symptom manifestation [40, 46]. Waiting for a consensus on a standardized methodology for an objective clinical assessment of electro-sensitivity, our present work referred to self-reported EHS as registered in the course of the anamnestic evaluation performed by trained medical personnel.

Data concerning the involvement of organic causes connected with chronic oxidative damage as a key factor in the induction and perpetuating of symptoms in functional SRI syndromes has been growing in the last decade (reviewed in: [22]). Our previous studies provided evidence of a specific and peculiar metabolic disease-marker profile in multiple chemical sensitivity, the prototype of all medically unexplained environmental illnesses so far described. In fact, moving from published data accounting for the altered redox balance in favor of a prooxidative and proinflammatory state in patients with fibromyalgia or chronic fatigue symptoms [7, 22], we identified a profile of 12 specifically altered blood parameters connected with systemic oxidative stress and impaired detoxification, in a representative sample of the Italian population fully or partially complying with MCS diagnosis [23]. In the same line, the present study was conceived to verify if analogous alterations of this pattern of MCS reliable organic biomarkers may also apply to EHS condition, in order to seek evidences of the organic etiology of this group of environmental sensitivity disorders and provide the clinicians with suitable tools for laboratory diagnosis and treatment follow-up.

The profiles of metabolic parameters' alteration observed in EHS subjects were comparable to those of the “pure MCS” group, though generally less pronounced (Figures 5–8). Similarly to those MCS patients self-reportedly nonelectrosensitive, the EHS cohort showed a highly significant-versus-control decrease in the erythrocyte GST activity and an increase in GPX activity levels (Figure 5), coupled with a marked decrease of GSH levels (Figure 6). Again in line with MCS, EHS group showed a trend to the increase in erythrocyte CuZnSOD activity and to the depletion of the main lipophilic antioxidants in plasma-reduced coenzyme Q_10_ and alpha-tocopherol (vitamin E) (Figures 5 and 6). The most striking difference between the two patient subgroups was recorded, instead, for erythrocyte catalase. Enzymatic activity was in fact only slightly and not significantly, reduced in EHS as compared to control values, while the highly significant (*P* < 0.0001) reduction recorded in the MCS group (Figure 5) confirmed our previous reports, validating the relevance and selectivity of this blood metabolic marker specifically for the MCS condition [23], being previously confirmed also in those patients only partially complying with MCS criteria (suspected MCS group).

We also calculated the ratios between oxidized and reduced forms of glutathione and coenzyme Q_10_ as suitable indicators of a systemic oxidative and proinflammatory* status* [47]. Relative oxidation of the two redox molecules was increased, though not significantly, in both EHS and MCS groups versus CTR (Figure 6). Interestingly, only in electrosensitive subjects, the oxidized/total CoQ_10_ ratio reached statistical significance (*P* < 0.001) versus normal values. Due to its marked lipophilicity, coenzyme Q_10_ is essential, along with alpha-tocopherol and squalene, for skin protection against oxidizing environmental physicochemical stressors, and it is able to efficiently reach the skin from the blood compartment [48, 49]. The elevated oxidation of plasma coenzyme Q_10_ observed in EHS appears to be consistent with the higher frequency of cutaneous involvement in EHS (40.7%) symptoms self-reported by our experimental group (Figure 3(a)), as compared to the minor relative clinical relevance assessed in the classical MCS condition, previously described [23]. Accordingly, Figure 4 shows how the prevalent skin symptom, in the EHS but not in the MCS cohort, resulted in being acute or chronic dermatitis (eczema), a group of inflammatory skin diseases where systemic and local lipophilic antioxidant depletion is strongly implicated [48].

A second parameter proved to be significantly different (*P* < 0.05) between EHS and MCS groups that is the ratio omega-6/omega-3 polyunsaturated fatty acids in the erythrocyte membrane phospholipid fraction (Figure 7(c)). The ratio showed a remarkable elevation versus CTR in favor of the more proinflammatory *ω*6 PUFA in the MCS group (*P* < 0.001), while EHS values were instead nearly overlapping CTR values, data that appears consistent with the overall less pronounced prooxidative and proinflammatory state evidenced in EHS versus MCS, from the whole pattern of redox parameters investigated in this study. Again, this molecular marker difference between the two environmental hypersensitivities can possibly be connected with the clinical setting, where, for example, a higher frequency of pathological obesity with metabolic syndrome is observed in MCS [50], whereas EHS condition features a milder chronic inflammatory* status* [51].

As a whole, MCS values of all metabolic parameters studied confirmed our previous results obtained in a larger cohort of 226 MCS + sMCS patients [23], highlighting the reliability of the selected redox-marker panel on this additional study cohort. With two exceptions, (a) erythrocyte CuZnSOD activity, now found significantly increased (*P* < 0.0001) in MCS versus CTR (Figure 5(c)) whilst nonsignificant in the first study, and (b) plasma nitrites/nitrates values, significantly elevated in the previous study MCS cohort [23], a finding not confirmed in the present study (data not shown). These differences may possibly be related to the extreme individual genetic and metabolic variability characterizing MCS populations, even within the same ethnic, geographic, lifestyle, and cultural setting, which represented one of the difficulties facing SRI human studies [52].

The question as to whether genetic background may determine a proneness to environmental hypersensitive syndromes remains still unanswered, from the time of the first pioneer studies on multiple chemical sensitivity [53, 54], followed by a wealth of extensive investigations on MCS, FM, and CFS western populations worldwide [19, 23, 55]. We attempted to contribute to this unresolved issue of utmost relevance for diagnostic purposes in these poorly defined clinical settings. In previous works, we had investigated gene and allele frequencies of selected polymorphisms of a wide array of phase I and II xeno- and endobiotic metabolizing enzymes, GST (M1, T1 and P1), UDP-glucuronosyl transferase (UGT), and cytochrome P450 (CYP) variants belonging to the CYP2C9, CYP2C19, CYP2D6, and CYP3A5*3 isoenzymes. After a first study not showing any significant prevalence of the studied CYP, UGT, and GST gene polymorphisms in a group of 110 MCS patients [23], we proceeded to a second investigation on a clinically better characterized MCS group of 156 patients and of 113 matched controls, where we identified significantly (*P* < 0.05–0.0001) higher frequencies versus CTR for the polymorphisms CYP2C9^*∗*^2, CYP2C9^*∗*^3, CYP2C19^*∗*^2, CYP2D6^*∗*^4, and CYP2D6^*∗*^41, confirming other studies indicating these genetic variants as a risk factor for SRI [24]. Starting from these results, in the present study, genotyping for the CYP2C19 single nucleotide variants showed that the frequency of the homozygous mutated *1 allele was significantly higher in EHS, than in MCS cases, whilst the *2 allele in the homozygous and heterozygous forms was less frequent in EHS than in MCS (*P* = 0.003) (Table 1). Moreover, our previous work had shown that the CYP2C19*2 heterozygous genotype *1/*2 was significantly more frequent (*P* = 0.05) in MCS cases, not only versus controls but also versus FM + CFS cases [24]. The same study showed for the first time that the Arg554Lys mutated variant of the aryl hydrocarbon receptor-AHR gene did not reach significant differences in distribution between SRIs and controls when analyzed alone but showed in specific haplotype combinations with CYP variants promising implications for in-between group discrimination within SRI comorbidities, namely, MCS versus sMCS and FC + FM versus controls [24]. In the present work, we were able to confirm the absence of significant differences for AHR genotype between EHS and CTR groups (data not shown).

Having previously found no significant difference between MCS patients and controls, in the distribution of GST isoenzyme genotypes [23], in the GST study we now compared EHS and healthy controls. Differently from our previous results on MCS, we here identified a mutated (null) allele combination of GSTT1 and GSTM1 variants able to predict risk of developing EHS by a 9.7 fold versus CTR (Table 1).

Taken together, our genetic results obtained on a number of cases due to be enlarged in the studies to come, although being far to be conclusive on such a controversial matter, can at least contribute additional indications to the complex mosaic of genetic risk factors in environmental hypersensitivities, still waiting to be correlated with individual metabolic phenotypes.

The outcomes of this work confirmed, in the whole, our previous results on MCS and provided additional evidences for the validity of the selected panel of metabolic blood parameters also in the self-reported EHS condition. Further developments must necessarily include a more objective and standardized classification of individual electromagnetic sensitivity scores, to conclusively assess the proposed parameters as a distinctive and specific panel of disease biomarkers for EHS. Our findings will hopefully contribute, in combination with the so-far putative genetic-risk factors, a better molecular definition of environmental-borne sensitivity-related illnesses and a tool to discriminate single SRI comorbidities, based on sufficiently proven molecular evidences able to gain clinical consensus.

## Figures and Tables

**Table 1:** Statistical analysis of genotype distribution of cytochrome P450 (CYP) isoenzymes in EHS-patients self-reporting electromagnetic hypersensitivity (*n* = 29) versus MCS-multiple chemical sensitivity patients without EHS (*n* = 85) and of glutathione S-transferase P1 (GSTP1), glutathione S-transferase M1 (GSTM1), and glutathione S-transferase T1 (GSTT1) isoenzymes in CTR-healthy control subjects (*n* = 68) versus EHS-patients (*n* = 127).

Genotype	CTR	EHS	MCS	*P*	Odds Ratio	C.I. 95%
CYP2C19 (*1/*1)		26 (89.7%)	51 (60.0%)	0.003		
CYP2C19 (*1/*2, *2/*2)		3 (6.9%)	34 (38.8%)			
GSTP1 (*A/*A, *A/*B)	62 (91%)	104 (82%)		0.09 n.s.
GSTP1 (*B/*B, *B/*C, *C/*C, *A/*C)	6 (9%)	23 (18%)		
GSTM1 (*1/*1, *1/*0)	36 (53%)	64 (50%)		n.s.
GSTM1 (*0/*0)	32 (47%)	63 (50%)		
GSTT1 (*1/*1, *1/*0)	58 (85%)	101 (80%)		n.s.
GSTT1 (*0/*0)	10 (15%)	26 (20%)		
GSTM1 (*1/*1, *1/*0) + GSTT1 (*1/*1, *1/*0)	67 (98.5%)	111 (87%)		0.007	9.7	(1.3–74.5)
GSTM1 (*0/*0) + GSTT1 (*0/*0)	1 (1.5%)	16 (13%)				
